# Evaluation of intracranial stenting in a simulated training and assessment environment for neuroendovascular procedures

**DOI:** 10.3389/fneur.2023.1247421

**Published:** 2023-09-04

**Authors:** Anna A. Kyselyova, Andreas M. Frölich, Maxim Bester, Caspar Brekenfeld, Jan-Hendrik Buhk, Andreas Ding, Frank Nagl, Tobias J. Jost, Helena Guerreiro, Ngoc Tuan Ngo, Jens Fiehler, Fabian Flottmann

**Affiliations:** ^1^Department of Diagnostic and Interventional Neuroradiology, University Medical Center Hamburg-Eppendorf, Hamburg, Germany; ^2^Department of Neuroradiology, Asklepios Hospitals St. Georg and Wandsbek, Hamburg, Germany; ^3^Acandis, Acandis GmbH & Co. KG, Pforzheim, Germany

**Keywords:** stroke, intracranial stenting, stenosis, simulation, flow model

## Abstract

**Purpose:**

Given the inherent complexity of neurointerventional procedures and the associated risks of ionizing radiation exposure, it is crucial to prioritize ongoing training and improve safety protocols. The aim of this study is to assess a training and evaluation *in-vitro* environment using a vascular model of M1 stenosis, within a clinical angiography suite, without relying on animal models or X-ray radiation.

**Materials and methods:**

Using a transparent model replicating M1 stenosis, we conducted intracranial stenting procedures with four different setups (Gateway & Wingspan, Gateway & Enterprise, Neurospeed & Acclino, and Pharos Vitesse). A video camera was integrated with the angiography system’s monitor for real-time visualization, while a foot switch was employed to simulate live fluoroscopy. Three neuroradiologists with varying levels of expertise performed each procedure for three times. The total duration of fluoroscopy as well as the time from passing the stenosis with the wire to completion of the procedure were recorded using a dedicated software designed for this experimental setup.

**Results:**

Compared to the Gateway & Wingspan procedure, the total fluoroscopy time reduced significantly with the Gateway & Enterprise, Neurospeed & Acclino, and Pharos Vitesse procedures by 51.56 s, 111.33 s, and 144.89 s, respectively (*p* < 0.001). Additionally, physicians with under 2 years and over 5 years of experience reduced FT by 62.83 s and 106.42 s, respectively, (*p* < 0.001), compared to a novice physician. Similar trends were noted for the time of wire distal to stenosis, with significant reductions for Neurospeed & Acclino and Pharos Vitesse compared to both Gateway & Wingspan as well as Gateway & Enterprise (all *p* < 0.001).

**Conclusion:**

Procedures requiring wire exchange maneuvers exhibited nearly twice the fluoroscopy time in comparison to balloon-mounted stenting or stent-placement via PTA balloon catheters. The more experienced neuroradiologist demonstrated significantly quicker performance in line with expectations in a real-life clinical setting, when compared to the less experienced interventionalist. This *in-vitro* setup allowed the evaluation of alternative technical approaches and differences in experience of operators without the use of animal models or X-ray. The setup combines advantages of simulators and silicone vessel models in a realistic working environment.

## Introduction

Stroke remains a leading cause of mortality and disability worldwide (1). Many clinical studies have shown the indisputable value of interventional stroke treatment (2–5). Intracranial atherosclerotic stenosis is one of the main factors of symptomatic ischemic stroke within the corresponding vascular territory (6). The severity of intracranial vessel stenosis, as evidenced by the WASID trial, exhibits a progressive association with the likelihood of recurrent ischemic stroke (7). Notably, the SAMMPRIS trial has revealed a substantial risk of neurological complications associated with intracranial angioplasty and stenting, reporting a 30-day stroke or death rate of 14.7% within the percutaneous transluminal angioplasty and stenting group (8). Similar results have been shown in the VISSIT trial (9). Therefore, the indication for this treatment has been substantially limited to a certain group of patients. Nonetheless, its implementation remains essential, particularly in acute or progressive stroke scenarios (10–13).

This increase in performed neurointerventional treatments leads to a high necessity of sufficient training. Due to improved imaging possibilities the numbers of diagnostic angiographies are decreasing, thereby diminishing opportunities for acquiring fundamental catheter manipulation skills during clinical practice (14). In addition, neurointerventional procedures are highly specialized and are typically performed in dedicated centers, thereby limiting both the case volume and clinical exposure for young professionals (15).

Due to the delicate nature of the nervous vessel system, neurointerventions require thorough preparation and training to minimize the risk of errors. The growing demand for efficient and cost-effective healthcare services has highlighted the need for educational technologies and simulation devices that ensure patient safety. These include computer-based simulators, plastic or silicone vessel models, and animal models.

The use of animal models in neurointerventional training presents both ethical concerns and limitations in reproducing human anatomy accurately (16, 17). Computer-based simulators show a great number of trained set-ups but lack a realistic haptic feeling of the used devices and interactions with the human vasculature.

An additional drawback of several realistic *in-vitro* training setups is their reliance on X-ray imaging, which restricts the training opportunities for specific neurointerventional specialists. Moreover, it contributes to an accumulation of X-ray exposure among these specialists, posing potential health risks.

In our study, we conducted an evaluation of a training setup designed to integrate the benefits of silicone-model-based and computer-based simulation methods within a realistic Cath-lab environment, eliminating the need for X-ray imaging. We specifically focused on the technical variations of endovascular treatment for intracranial stenosis, considering it an appropriate scenario for examining the efficacy of the *in-vitro* training setup.

Our hypothesis posits that the *in-vitro* environment enables the assessment of procedure times, taking into account the expertise of the neurointerventionalist and the complexity of the intervention.

## Materials and methods

### Model

The vascular model utilized in this study incorporates a combination of a simplified delivery pathway and a modified patient-specific anatomy model. The delivery pathway consists of a silicone tube (50 cm long, with an inner diameter of 0.8 cm) mounted on an acrylic plate. It is proximally connected to a hemostasis valve with continuous flushing for access purposes. The distally connected patient-specific segment includes a translucent silicone tube bifurcation model representing the cervical section (10 cm in length, with inner diameters of 0.8 cm for the Common Carotid Artery, and 0.5 cm for the Internal Carotid Artery (ICA)). A vessel model of the intracranial ICA and M1 segment with a stenosis was produced by additive manufacturing using a commercially available 3D printer (Form 2, Formlabs, Somerville, MA, USA) and a flexible resin (Flexible 80A, Formlabs, Somerville, MA) (18). To facilitate smooth catheter movements within the model, a continuous flow of water tempered to 37° Celsius, and containing a commercially available soap as a surfactant substance, is utilized.

**Figure 1:**
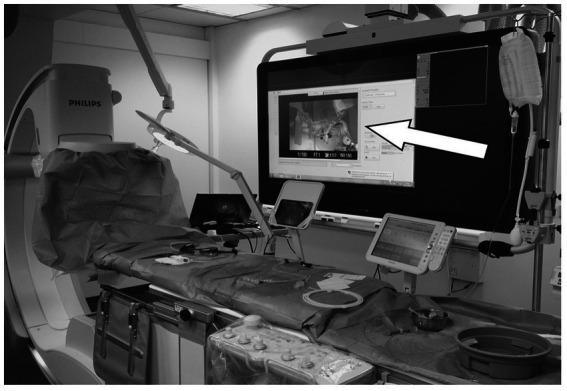
Interventional neuroradiological Angio Suite Allura Clarity FD 20 (Philips Healthcare) with the training system set up (arrow shows the camera recording of the vessel model).

This model setup is placed on the table of a clinical angiography system (Allura Clarity FD 20, Philips Healthcare, Best, The Netherlands). To make sure that visual control for the operator is exclusively possible upon activating the foot switch the stenosis model is covered with a drape (see Figure 1).

Fluoroscopy, which mimics real-time imaging, is simulated through a foot switch that is connected to the camera system. This allows live visualization of the target zone, and upon turning off the foot switch, the image is frozen, simulating the “last image hold” feature commonly available in clinical angiography systems. This allows a precise determination of fluoroscopy time in a simulated real life clinical scenario. To replicate a realistic treatment situation, the camera is connected to the large multipurpose monitor of the angiography system (Flexvision, Philips Healthcare, Best, The Netherlands, see Figure 1).

### Procedural simulation

To ensure the avoidance of unrealistic conditions in the proximal tubes, all procedures in this study were conducted using a 6F Envoy guiding catheter (Codman Neuro, Raynham, MA), which was placed in the internal carotid artery. The start of the procedure was defined as insertion of the first microdevice into the guiding catheter, and the end of the procedure was defined as removal of the last microdevice.

To account for varying levels of expertise, all procedures were performed three times by three physicians with different experience levels, following the same predetermined sequence. Physician #1 had no prior experience in neurointerventional radiology (INR), physician #2 had less than 2 years of experience in INR, and physician #3 had over 5 years of experience in INR.

The study involved simulating four different variants of treating an M1 stenosis, all adhering to the instructions for use (IFU) specific to the materials employed.

1. “Gateway & Wingspan”: The stenosis was initially dilated using a Gateway™ percutaneous transluminal angioplasty (PTA) balloon (Stryker Neurovascular, Kalamazoo, MI, USA), which was advanced over a 300 cm Synchro™ 0.014″ microwire (Stryker Neurovascular, Kalamazoo, MI, USA). Following the predilation, the PTA balloon was replaced by a Wingspan Stent Delivery System (Stryker Neurovascular, Kalamazoo, MI, USA), utilizing the long microwire, and the stent was deployed. Subsequently, all microdevices were withdrawn. This method served as standard of reference since it only includes material applied fully in line with the official authorization and its instructions for use.
2. “Gateway & Enterprise”: The stenosis was initially dilated using a Gateway™ PTA balloon, which was advanced over a 300 cm Synchro™ 0.014″ microwire. Subsequently, the PTA balloon was exchanged via the long microwire into a Prowler Select Plus™ 0.021″ microcatheter (Codman Neurovascular, Raynham, MA, USA), allowing it to cross the stenosis. An Enterprise™ stent (Cerenovus, Fremont, CA, USA) was introduced into the microcatheter and then deployed at the stenosis site. Finally, all microdevices were withdrawn. This method served as example for exchanging to a catheter-delivered stent after the initial PTA.
3. “Neurospeed & Credo”: The stenosis was initially dilated using a Neurospeed™ percutaneous transluminal angioplasty (PTA) balloon (Acandis GmbH, Pforzheim, Germany), which was advanced over a 200 cm Synchro™ 0.014″ microwire. Following the predilation, a Credo stent (Acandis GmbH & Co. KG, Pforzheim, Germany) was introduced into the central lumen of the PTA balloon catheter. The deployment of the stent was performed by expanding it over the PTA balloon at the level of the stenosis. Finally, all microdevices were withdrawn. This method served as an example in the study to illustrate the approach of delivering a stent through the inner lumen of the PTA balloon.
4. “Pharos Vitesse”: The stenosis was directly crossed using a Pharos Vitesse™ balloon-expandable stent system (Codman Neurovascular, Raynham, MA, USA) over a 200 cm Synchro™ 0.014″ microwire. After dilatation the stent was deployed and micromaterials were withdrawn.
  Following dilatation, the stent was deployed, and all microdevices were withdrawn. This method served as an example in the study to demonstrate the utilization of a balloon-mounted stent. It is important to note that the use of the Pharos Vitesse™ stent is generally considered off-label, as the stent itself has been withdrawn from the market.

### Data acquisition

Fluoroscopy time during the procedures was measured using a dedicated, non-commercial software that operated through a footswitch and camera system. To obtain additional time data for specific procedural steps, the software recorded time stamps for the following events: when the microwire reached its final position (passage through the stenosis), during inflation and deflation of devices, if applicable, insertion of the stent delivery system, if applicable, stent positioning, if applicable, stent deployment, and finally, the removal of the wire, indicating the end of the procedure.

For statistical analysis, we selected the duration of the microwire located distal to the stenosis (WDST) as a surrogate parameter. This choice allowed us to differentiate between the various treatment concepts tested and compare their respective durations.

### Statistical analysis

Statistical analyses were performed using R software version 4.2.2.

For the analysis of fluoroscopy time, a multiple linear regression was conducted with Procedure, Physician, and Trial as predictors, using the “Gateway & Wingspan” procedure, novice physician and trial 1 as the standard of reference. The assumptions of linearity, independence, homoscedasticity, normality, and absence of multicollinearity were assessed. Linearity was examined visually using a plot of observed versus predicted values. Independence of residuals was assessed using the Durbin-Watson test. Homoscedasticity was tested using the Breusch-Pagan test. Normality of residuals was checked with the Anderson-Darling test. Multicollinearity was evaluated using the Variance Inflation Factor (VIF).

For the analysis of WDST, because the assumption of independence was not met in the preliminary linear regression model (Durbin-Watson test, DW = 1.33, *p* = 0.004), a one-way ANOVA was conducted on the log-transformed WDST data with Procedure as the predictor. Normality of residuals was assessed using the Anderson-Darling test, and homogeneity of variances was evaluated with Levene’s test. When significant differences were found in the ANOVA, *post hoc* comparisons were performed using Tukey’s HSD test.

A value of *p* of less than 0.05 was considered statistically significant.

## Results

Each procedure was performed by three physicians in three different trials, and the results for total fluoroscopy time (FT) and time of wire distal to stenosis (WDST) were collected (Table 1).

**Table 1:** The fluoroscopy time (FT) and wire distal to stenosis time (WDST) for various simulated techniques (physician #1: no experience, physician #2: <2 years and physician #3: > 5 years of experience in INR).

Procedures		Gateway & Wingspan		Gateway & Enterprise		NeuroSpeed & Credo		Pharos Vitesse	
		FT (s)	WDST (s)	FT (s)	WDST (s)	FT (s)	WDST (s)	FT (s)	WDST (s)
Physician #1	Trial 1	257	320	210	244	227	85	95	50
	Trial 2	305	358	226	229	138	61	104	49
	Trial 3	385	398	211	231	173	81	88	34
Physician #2	Trial 1	184	240	160	190	100	51	75	52
	Trial 2	216	284	189	198	88	39	86	71
	Trial 3	224	284	188	199	79	45	76	57
Physician #3	Trial 1	136	171	132	122	86	31	32	21
	Trial 2	149	192	109	142	52	15	78	51
	Trial 3	143	172	110	119	54	33	61	40
	Mean	222	269	171	186	111	49	77	47
	SD	78	77	42	45	55	22	24	11

**Figure 2:**
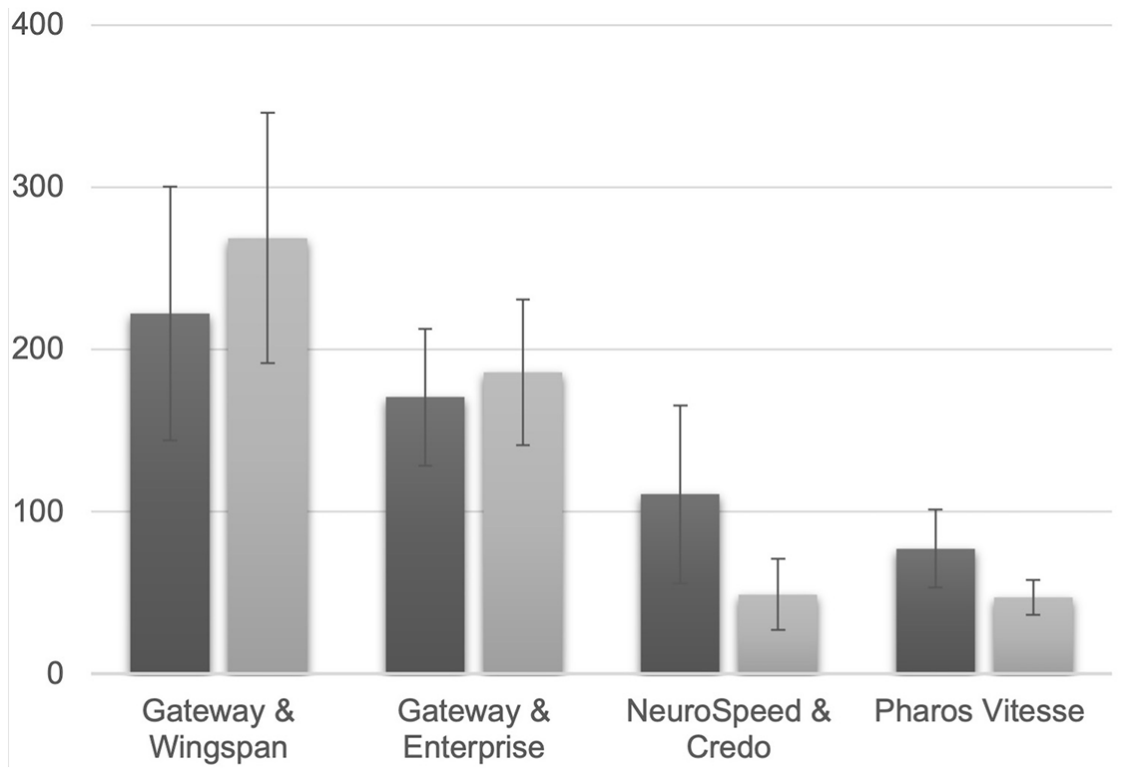
The average fluoroscopy time (first bar) and average wire distal to stenosis time (second bar) for various techniques. The values are measured in seconds (s).

The mean values for total fluoroscopy time (FT, s) and time of wire distal to stenosis (WDST, s) values, along with their standard deviations, were calculated for each procedure and physician (Figure 2). The average FT times ranged from 77 s (Pharos Vitesse) to 222 s (Gateway & Wingspan), and the mean WDST times varied between 47 s (Pharos Vitesse) to 269 s (Gateway & Wingspan).

In multiple linear regression analysis, the different procedures and physicians were significantly associated with the total fluoroscopy time (FT), while the trials were not (see Table 2). Relative to the reference (Gateway & Wingspan and Physician #1 and Trial 1), the procedures Gateway & Enterprise, Neurospeed & Acclino, and Pharos Vitesse significantly reduced the FT times by 51.56 s, 111.33 s, and 144.89 s, respectively (all *p* < 0.001). Additionally, when procedures were performed by a physician with some experience (<2 years) and a physician with more experience (>5 years), the FT times significantly decreased by 62.83 s and 106.42 s (both *p* < 0.001), compared to a physician with no experience. However, no significant effect was found for the second and third trials on FT times (*p* = 0.79 and *p* = 0.56, respectively).

**Table 2:** Summary of coefficients in the linear regression model for fluoroscopy time using various techniques, compared to the Gateway & Wingspan reference and considering the experience levels of the operators (physician #1: no experience, physician #2: <2 years and physician #3: > 5 years of experience in INR) and trial number.

FT, s	Estimate	Std. error	*t*-value	Value of *p*
Intercept	274.53	16.09	17.07	<0.001
Gateway & Enterprise	−51.56	16.09	−3.20	<0.001
Neurospeed & Acclino	−111.33	16.09	−6.92	<0.001
Pharos Vitesse	−144.89	16.09	−9.01	<0.001
Physician #2	−62.83	13.93	−4.51	<0.001
Physician #3	−106.42	13.93	−7.64	<0.001
Trial T2	3.83	13.93	0.28	0.79
Trial T3	8.17	13.93	0.59	0.56

For the time of wire distal to stenosis (WDST), the one-way analysis of variance (ANOVA) revealed a significant effect of Procedure on the log-transformed WDST (*p* < 0.001). Pairwise comparisons using Tukey’s HSD test revealed that, compared to GatewayWingspan (mean WDST = 268 s), both NeurospeedAcclino (mean 49 s) and Pharos (mean 47 s) led to significantly lower log-transformed WDST values (value of *p* <0.001 for both). There was no significant difference between GatewayEnterprise (mean 186 s) and GatewayWingspan (value of *p* = 0.2169). Also, there was no significant difference between Pharos and NeurospeedAcclino (value of *p* = 0.9984).

## Discussion

By utilizing an X-ray-free *in-vitro* setup specifically designed for intracranial stenosis, we conducted an evaluation of procedural and fluoroscopy times associated with four different technical approaches to angioplasty. These procedures were performed by three neurointerventionalists with varying levels of experience. Our findings revealed that the technical approaches with lower complexity were completed more rapidly. Furthermore, more experienced neurointerventionalists exhibited lower fluoroscopy times during the procedures.

Simulation of neurointerventional procedures holds great significance in medical education and training. It serves as a valuable tool for various purposes, including crisis management training, enhancing perioperative communication skills, conducting assessments, and facilitating preoperative planning for challenging procedures (15, 19). The lack of statistical results in published data regarding interventional simulation models hinders the ability to compare these models with real-life clinical data (15). Moreover, it is essential for an adequate training environment to have the capability to evaluate intervention outcomes and procedure success, considering the experience level of the interventional specialist. In addition to physical models, neurointerventional procedures can also be practiced using commercially available virtual reality simulators. These simulators generate computer-simulated angiography-like images and allow control using specialized catheters and wires. This approach offers notable advantages, including high flexibility, a wide range of available procedures, and virtually unlimited repeatability. Furthermore, these simulators can be easily set up in various locations, unlike animal models that invariably require fluoroscopy.

However, the realism of haptic feedback in computer-based simulation is limited, particularly in critical situations such as implant and catheter dislocation, which may compromise the training experience. Additionally, the integration of specific devices into computer-based simulations is required, limiting the availability of interventional tools. In contrast, physical vascular models offer the advantage of utilizing actual catheter materials with realistic haptic feedback, enabling detailed investigations into catheter and implant behavior.

While the training scenario presented in this study may be less flexible compared to computer-based simulations in terms of case variety and portability, it combines the advantages of computer-based simulators with vessel model-based simulations. It provides physicians with a realistic environment that closely resembles a fluoroscopy workplace without the need for X-ray exposure. A significant advantage of this approach is the use of genuine devices and the ability to replicate individual vessel models, which can now be produced with exceptional quality using additive manufacturing techniques (18, 20). Moreover, the versatility of this system allows for its deployment in various settings, with the only limitation being the absence of a dedicated Cath lab environment, which would further enhance the realism experienced by participants.

In our study, we aimed to simulate a representative challenging procedure that encompasses different technical approaches. The collected data indicate that the setup successfully replicates the expected outcomes. Specifically, the Wingspan procedure, which necessitates an exchange maneuver, is known to have a relatively extended duration, and our results align with this expectation (21, 22). On the contrary, the deployment of balloon-mounted stents can be accomplished rapidly with a single inflation; nevertheless, there may be challenges related to access (22, 23). The utilization of the PTA balloon for the placement of the intracranial stent appears to be a viable approach, as it eliminates the need for an exchange maneuver and significantly reduces fluoroscopy time, as it is shown in our study. The observed differences between experienced and non-experienced operators align with findings from previous studies as well (24, 25). The absence of significant difference in fluoroscopy time and procedure duration between trial two and trial three, when compared to the initial attempt, could be attributed to the repetitive nature of the trial setting itself.

Due to the absence of radiation exposure in the presented scenario, it offers high flexibility in terms of the target audience. It can be incorporated into training courses for a wide range of participants, including specialists, residents, students, technicians, and nurses.

To address the decline in physician’s knowledge over time and ensure the maintenance of high standards and patient safety, several certification programs have been implemented, such as Continuous Professional Development (CPD), Maintenance of Certification (MOC), and Practice Quality Improvement (PQI) (19). These programs aim to promote ongoing learning, professional development, and quality improvement in medical practice. The utilization of a written test as a sole form of knowledge evaluation is inadequate for comprehensively assessing neuroradiological and neurointerventional skills. In this context, the presented set-up provides an illustrative example of an objective and quantitative performance assessment method for neurointerventional trainees. Additionally, it facilitates the evaluation of skill enhancement and development over a period, ensuring a more comprehensive evaluation of interventional capabilities.

## Limitations

This set-up specifically focuses on the intracranial access and stent deployment steps, omitting the preceding challenging aspects encountered in real clinical situations. By simulating stent deployment, certain complications, such as dislocation, can be adequately assessed and practiced within this framework. However, as with all *in-vitro* models, our model is limited in its ability to replicate real blood flow and reactive vasculature, thereby preventing the occurrence of complications such as rupture, dissection, thrombosis, and others, which are also crucial to be trained and assessed. To address this limitation, future advancements could involve the development of complication-based *in vitro* models with more fragile vasculature.

Moreover, our assessment of procedure quality relied on fluoroscopy and procedure times as surrogate markers, without directly evaluating the positioning of the stent, remaining stenosis, and re-occlusion. It is important to note that the inclusion of an evaluation form or a suitable questionnaire could enhance the integration of such a training environment within an educational curriculum and assessment framework.

## Conclusion

Our study presents and evaluates a cost-effective, animal-free, and X-ray-free model for endovascular training for the treatment of intracranial stenosis. This training setup combines the benefits of computer-based and flow-model-based simulation, making it suitable for integration into an educational curriculum for neuroendovascular procedures. The use of this model allows for the measurement and evaluation of the training level and improvement of interventional skills within a realistic working environment.

## Data availability statement

The original contributions presented in the study are included in the article/supplementary material, further inquiries can be directed to the corresponding author.

## Ethics statement

Ethical review and approval was not required for the study on human participants in accordance with the local legislation and institutional requirements. Written informed consent from the participants or participants legal guardian/next of kin was not required to participate in this study in accordance with the national legislation and the institutional requirements.

## Author contributions

J-HB: conception and design of work. AK, AF, J-HB, MB, CB, FN, and TJ: data collection. J-HB, AK, AD, NN, JF, and FF: data analysis and interpretation. J-HB, AK, and FF: drafting the article. AK, AF, MB, CB, JF, J-HB, AD, FN, TJ, HG, NN, and FF: critical revision of the article. JF and FF approved the final version to be published. All authors contributed to the article and approved the submitted version.

## Conflict of interest

J-HB received personal fees from Consultant for Microvention, Stryker, Cerenovus, Acandis, and Medtronic outside the submitted work. JF received personal fees from Consultant for Microvention, Stryker, Cerenovus, Acandis, Penumbra, and Medtronic outside the submitted work. He is a member of the Executive Board of the scientific societies DGNR and ESMINT. AD, FN, and TJ were employed by Acandis, Acandis GmbH & Co. KG.

The remaining authors declare that the research was conducted in the absence of any commercial or financial relationships that could be construed as a potential conflict of interest.

## Publisher’s note

All claims expressed in this article are solely those of the authors and do not necessarily represent those of their affiliated organizations, or those of the publisher, the editors and the reviewers. Any product that may be evaluated in this article, or claim that may be made by its manufacturer, is not guaranteed or endorsed by the publisher.
